# Liver dysfunction after lung recruitment manoeuvres during pressure-controlled ventilation in experimental acute respiratory distress

**DOI:** 10.1186/cc5674

**Published:** 2007-01-29

**Authors:** Markus Kredel, Ralf M Muellenbach, Robert W Brock, Hans-Hinrich Wilckens, Joerg Brederlau, Norbert Roewer, Christian Wunder

**Affiliations:** 1 University of Würzburg, Department of Anaesthesiology, University Hospital Würzburg, Oberdürrbacherstr. 6, 97080 Würzburg, Germany; 2 University of Arkansas for Medical Sciences, Department of Pharmacology and Toxicology, 4301 Markham St., Little Rock, AR, USA

## Abstract

**Introduction:**

Consequences of lung recruitment with prolonged high positive end-expiratory pressure (PEEP) ventilation for liver function are unclear. We therefore investigated liver dysfunction during two different ventilation treatment regimens of experimental acute respiratory distress syndrome.

**Methods:**

Sixteen anaesthetised juvenile pietrain pigs were ventilated in the pressure-controlled mode (PCV) with an inspiratory fraction of oxygen (FiO_2_) of 1.0, a respiratory frequency of 30 per minute, a tidal volume of 6 ml/kg, and a PEEP of 5 cm H_2_O. After lung injury was induced by repeated pulmonary lavage with normal saline, animals were randomly assigned into two groups (*n *= 8 each) for a 24-hour trial: PCV (unchanged ventilation) and PCV with recruitment (PCV+R) (starting with a sustained inflation of 50 cm H_2_O for 1 minute, the ventilation was continued while increasing PEEP in increments of 3 cm H_2_O every 15 minutes as long as arterial oxygen tension [PaO_2_] improved). After recruitment, FiO_2 _was reduced to 0.4 and the PEEP was lowered every 15 minutes until PaO_2 _decreased to 12.0 to 14.7 kPa (90 to 110 torr). Serum levels of hyaluronic acid (HA), routine liver serum markers, and plasma disappearance rate of indocyanine green (ICG) were tested before and after lung injury, and 6 and 18 hours after randomisation. Liver serum markers were also tested at 24 hours. Paraffin sections of liver tissue stained by haematoxylin and eosin were made after euthanisation.

**Results:**

The PCV+R group exhibited more polymorphonuclear neutrophils and lymphocytes in the liver sinusoids: median score (interquartile range) of 1.5 (1.4 to 1.5) compared to 0.9 (0.7 to 1.1) (*p *= 0.01). Elevation of bilirubin, aspartate aminotransferase, and lactate dehydrogenase was more prominent in the PCV+R group. Plasma disappearance rate of ICG indicated no liver dysfunction. HA levels in the PCV+R group gradually increased and were significantly higher (*p *< 0.001) at 6 and 18 hours with 59 (57 to 64) and 75 (66 to 84) ng/ml, respectively, than in the PCV group with 34 (32 to 48) and 41 (38 to 42) ng/ml, respectively.

**Conclusion:**

The PCV+R group showed a more prominent inflammatory reaction in their liver sinusoids accompanied by increased serum levels of liver enzymes and HA. Therefore, recruitment with higher PEEP levels for treatment of respiratory failure might lead to liver dysfunction.

## Introduction

Most patients with acute respiratory distress syndrome (ARDS) die not from hypoxaemia but from multiple organ failure [1]. Protective ventilation strategies using low tidal volumes do not necessarily improve oxygenation immediately but ultimately increase survival [2]. Accordingly, multiple organ failure may be a consequence not only of the underlying disease or ARDS itself but of the mode of artificial ventilation applied [3].

Recruitment manoeuvres and high levels of positive end-expiratory pressure (PEEP) are expected to ameliorate the biotrauma of the lung by preventing repetitive collapse and reopening atelectatic lung regions. However, the addition of higher PEEP levels to low tidal volume ventilation had no influence on survival in ARDS despite better oxygenation [4]. High PEEP levels reduce hepatic venous outflow [5] and splanchnic oxygen content difference [6]. Therefore, ventilation regimens using high airway pressure affect liver function, and this may have consequences for the clinical outcome of patients.

Several tests are capable of detecting different aspects of liver damage or impaired liver function. Besides measurements of liver cell enzyme plasma levels and histopathologic examination (which are used routinely), functional liver tests like plasma clearance of indocyanine green (ICG) are available. Increased plasma levels of hyaluronic acid (HA) are markers of acute [7] and chronic [8] liver damage. HA is produced in connective tissue cells found all over the body and is a component of the extracellular matrix [9]. A hallmark of tissue injury is increased turnover of HA. Small-molecular weight fragments of HA produced during systemic inflammation have been shown to activate dendritic cells and macrophages through nuclear factor-κB signaling [10]. Additionally, it has been shown recently that high-molecular weight HA provides a protective signal to lung epithelial cells during acute lung injury [11]. Thus, HA seems to play a double-edged role during inflammatory lung injury [12]. HA that reaches the systemic circulation is incorporated mainly by liver sinusoidal endothelial cells via a receptor-mediated mechanism [13,14]. Therefore, increase of plasma HA is now increasingly used as an indicator of liver sinusoidal endothelial cell dysfunction. It was shown recently that the inflammatory reaction in liver sinusoids, induced by bilateral hindlimb ischaemia reperfusion, was accompanied by an increase in plasma HA levels [15].

We proposed that pronounced high-pressure ventilation for treatment of experimental ARDS might induce liver damage. The diagnostic value of established methods to detect liver injury in this setting is unclear. Therefore, we chose two ventilatory strategies for using lung-protective pressure-controlled ventilation (PCV) with low tidal volumes. But in one group, a recruitment strategy was used (PCV+R): The lung was opened by a recruitment manoeuvre with initial sustained inflation and incremental PEEP trial achieving maximal oxygenation followed by reduction of the inspiratory fraction of oxygen (FiO_2_) and decrease of PEEP as long as arterial oxygen tension (PaO_2_) remained more than 12.0 kPa (90 torr). We performed serial measurements of liver tests in a 24-hour ventilatory period and a final histological examination of the liver.

## Materials and methods

### Animals and preparation

This study was conducted according to the German Animal Protection Act and was approved by the Laboratory Animal Care and Use Committee of the District of Unterfranken, Germany. Sixteen juvenile female pietrain pigs with body weight ± standard deviation of 53.2 ± 4.5 kg were fasted for 24 hours without limiting water access. After intramuscular sedation with 1 mg/kg (body weight) xylazine, 10 mg/kg *S*-ketamine, and 25 μg/kg atropine, peripheral venous access was obtained via an ear vein. Pigs were placed in a supine position, and anaesthesia was induced with 10 mg/kg thiopental.

Endotracheal intubation was performed with a cuffed 8.5-mm inner-diameter endotracheal tube with an additional pressure measurement lumen ending at the tip of the tube (Rüschelit^®^, Rüsch AG, Kernen, Germany). PCV was performed with a Servo^® ^900C ventilator (Siemens-Elema AB, Solna, Sweden). PEEP was set to 5 cm H_2_O and the peak inspiratory pressure was adjusted to achieve tidal volumes of 6 ml/kg body weight with a frequency of 30 per minute. FiO_2 _was 1.0 and inspiratory-to-expiratory time ratio was set to 1:1. Anaesthesia was maintained with thiopental (5 to 10 mg/kg per hour), fentanyl (10 μg/kg per hour), and pancuronium bromide (0.1 mg/kg per hour). Adequate hydration was maintained by continuous infusion of 4 ml/kg lactated balanced electrolyte solution throughout the experiment. Cefazoline (100 mg/kg) was administered intravenously every eight hours for prophylaxis of bacterial infection.

The left carotid artery of each pig was cannulated with an arterial catheter (Vygon, Ecouen, France), and a percutaneous introducer sheath (Arrow International, Inc., Reading, PA, USA) was inserted into the right internal jugular vein. A urinary bladder catheter was placed transcutaneously. All punctures were performed under ultrasonic guidance (SonoSite180 Plus^®^; SonoSite, Inc., Bothell, WA, USA). A pulmonary artery catheter (831F75; Edwards Lifesciences LLC, Irvine, CA, USA) was introduced under transduced pressure guidance through the sheath.

### Experimental protocol

After a 30-minute stabilisation period, baseline measurements (T_baseline_) were done. Lung injury was performed by repeated lavage (30 ml/kg) with prewarmed 38°C sterile 0.9% saline solution through the endotracheal tube for surfactant washout. Lavage fluid ran out passively and ventilation was continued applying PCV with 6 ml/kg tidal volume by adjusting the inspiratory pressure accordingly. Lavage was repeated every 10 minutes until PaO_2 _was less than 8.0 kPa (60 torr). Additional lavages were performed until PaO_2 _remained less than 8.0 kPa for at least one hour. Then, measurements were performed (T_ALI_) and each animal was randomly assigned to the PCV or the PCV+R group.

In the PCV group, ventilator settings remained unchanged throughout the 24-hour trial, except for the peak inspiratory pressure, which was adjusted throughout the experiment to maintain a tidal volume of 6 ml/kg. In the PCV+R group, a recruitment manoeuvre was performed after infusion of 500 ml of colloid solution (Voluven 6% HES 130/0.4; Fresenius Kabi AG, Bad Homburg, Germany). During the experiment, additional colloid solution was given in both groups if critical hypotension responding to volume replacement occurred. Sustained inflation of the lungs was performed applying 50 cm H_2_O for one minute or until severe compromise of blood pressure occurred. Afterward, ventilation was continued with a PEEP of 8 cm H_2_O. After an equilibration phase of 15 minutes, arterial blood gases were drawn and PEEP was increased in increments of 3 mm H_2_O every 15 minutes until PaO_2 _did not increase any more or until it decreased. Respiratory rate was adjusted up to 40 per minute if arterial carbon dioxide tension (PaCO_2_) increased to more than 6.0 kPa (45 torr) and no auto-PEEP of more than 1 cm H_2_O developed. After the maximum PaO_2 _was reached, the PEEP was reduced stepwise by 3 cm H_2_O and FiO_2 _was decreased stepwise to 0.4 until PaO_2 _remained stable within 12.0 to 14.7 kPa (90 to 110 torr). A repeated sustained inflation and PEEP enhancement by 3 cm H_2_O was performed whenever PaO_2 _decreased to less than 12.0 kPa.

### Measurements

Arterial and pulmonary artery catheter pressures were measured via pressure transducers (Exadyn-Combitrans^® ^Monitoring-Set; B. Braun Melsungen AG, Melsungen, Germany) zeroed at the mid-thorax level. Vascular pressures and electrocardiography were shown on a Servomed^® ^monitor (Hellige, Freiburg, Germany). Blood gases were analysed with an ABL 505 (Radiometer A/S, Brønshøj, Denmark). Cardiac output was measured as the mean of three measurements after injecting a bolus of 10 ml of ice-cold saline (Explorer^®^; Edwards Lifesciences LLC) randomly during different phases of the respiratory cycle. The core temperature, measured by the pulmonary artery catheter, was maintained at 38°C by regulating a heating pad. Airway pressures at the tip of the tube, capnography, and peripheral oxygen saturation were measured with a PM 8050 monitor (Dräger, Lübeck, Germany). Liver function was determined by the non-invasive ICG plasma clearance method (LiMON; PULSION Medical Systems AG, Munich, Germany) after injection of 0.25 mg/kg of ICG (ICG-PULSION^®^; PULSION Medical Systems AG) into the right atrium at T_baseline_, T_ALI_, T_6 h _(time point six hours after randomisation), and T_18 h_.

At T_baseline_, T_ALI_, T_6 h_, T_18 h_, and T_24 h_, serum samples were drawn for immediate determination of aspartate aminotransferase (AST), alanine aminotransferase (ALT), γ-glutamyltransferase (GGT), lactate dehydrogenase (LDH), alkaline phosphatase (AP), cholinesterase (ChE), total bilirubin, direct bilirubin, and lactate in the central clinical laboratory by using routine methods.

### HA assay

HA levels were determined out of serum samples drawn at T_baseline_, T_ALI_, T_6 h_, and T_18 h_. A standard enzyme linked protein-binding assay kit (Corgenix Medical Corporation, Broomfield, CO, USA) was used according to the manufacturer's instructions. Wells coated with HA-binding protein were incubated with serum samples diluted in reaction buffer for one hour and were then discarded. The wells were washed four times and a chromogenic substrate was added. Coloured reaction was stopped after 30 minutes by addition of sulphuric acid (0.36 N). An enzyme-linked immunosorbent assay reader (Sunrise; Tecan, Männedorf, Switzerland) measured the colour intensity at 450 nm. The concentration of the samples was calculated using a standard curve. Samples were run in triplicate and assay analysis was blinded.

### Liver histopathology

At the end of the experiment, 24 hours after randomisation (T_24 h_), the animals were euthanised by an overdose of thiopental and T61 (Intervet Deutschland GmbH, Unterschleißheim, Germany). Tissue samples of the left medial lobe of the liver, approximately 4 cm proximal from the edge, were taken and fixed in 10% formaldehyde solution. Samples were embedded in paraffin blocks. Slides were stained with haematoxylin and eosin. Two independent investigators, blinded to the group assignment, graded five histological lobules of every animal's liver. The extent of necrosis, haemorrhage, portal inflammation, and sinusoidal inflammation was separately graded as 0 (not present), 1 (less than or equal to 20% of the lobulus), 2 (more than 20% to less than or equal to 50%), or 3 (more than 50%). Mean scores of the grading of a total of 10 lobules were calculated for each animal. Additionally, 10 random high-power fields (hpf) out of the entire sinusoidal area, excluding septal parts or parts of the central vein, were examined in each animal. Polymorphonuclear neutrophils and lymphocytes were counted and expressed as the median number of cells per high-power fields.

### Statistical analysis

Because normal distribution was not satisfying for the data in all variables, data were shown as median with interquartile range (25% and 75% percentiles) and non-parametric tests were performed. SigmaStat for Windows version 2.03 (Systat Software, Inc., San Jose, CA, USA) was used for statistical analysis. Mann-Whitney rank sum test was used to compare data of the two groups. For comparison of a number of time points in either group, Friedman repeated measures analysis of variance on ranks with all pairwise multiple comparison procedures (Student-Newman-Keuls method) was used. Agreement of results in histopathological examinations between investigators was tested with Spearman rank order correlation. A *p *value of less than 0.05 was considered as significant.

## Results

**Figure 1:**
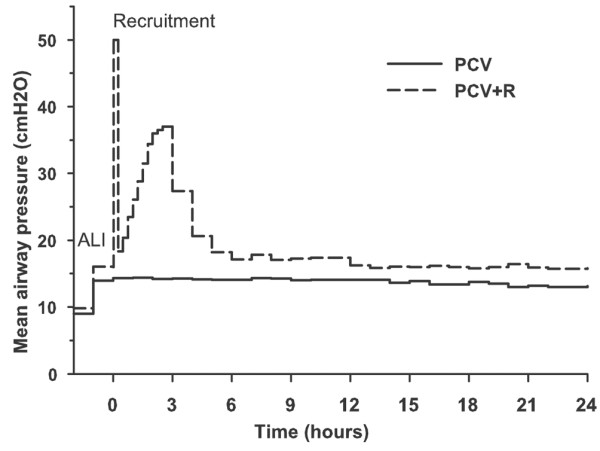
Mean airway pressure during the trial. After the induction of acute lung injury (ALI), a recruitment manoeuvre was performed in the PCV+R group by sustained inflation and stepwise increase of positive end-expiratory pressure (PEEP) by 3 cm H_2_O every 15 minutes. Recruitment was followed by stepwise reduction of PEEP according to target arterial oxygen tension. In the PCV group, level of PEEP remained at 5 cm H_2_O. Median values of the groups are shown. PCV, pressure-controlled ventilation; PCV+R, pressure-controlled ventilation with recruitment manoeuvre.

The number of saline lavages needed to induce lung injury was the same for the two groups: median of 17 (range 12 to 29) for the PCV group and median of 18 (range 26 to 14) for the PCV+R group. The course of the mean airway pressure for both study groups is shown in Figure 1. Recruitment with sustained inflation and stepwise PEEP increase was sustained in all animals of the PCV+R group without critical haemodynamic compromise, but cardiac output decreased from 6.8 (5.4 to 8.1) l/min at a PEEP of 5 cm H_2_O to 3.6 (3.2 to 3.8) l/min at a PEEP of 26 cm H_2_O, reached by all animals of this group 105 minutes after induction of lung injury. Meanwhile, mean arterial pressure decreased from 93 (81 to 101) to 75 (69 to 83) mm Hg. All but one animal survived the whole trial. After 21 hours, one pig of the PCV group was euthanised because fatal hypoxaemia was imminent. In this animal, haemodynamic measurements and blood samples drawn for liver enzyme levels in the 20th hour were used for the T_24 h _analysis. Electrolyte and colloid solution administration as well as urine output were not different between the two groups at the end of the experiment. No significant auto-PEEP (maximum of 1 cm H_2_O) was measured in the animals at any point during the trial.

Table 1 shows haemodynamic variables during the 24-hour trial. Baseline values were similar in the two groups. Heart rate was not different between the time points and groups. Mean arterial pressure was higher in the PCV+R group at T_ALI _despite randomisation; this was followed by a decrease during subsequent time points. Comparable changes in mean arterial pressure were also observed in the PCV group but were not significant. Central venous pressure at T_baseline _in the PCV+R group was lower than at later measurements but was not different from the PCV group. Cardiac output was significantly higher at T_ALI _in the PCV+R group despite randomisation but lower at T_6 h _than in the PCV group. In the PCV group, cardiac output remained stable but began to decrease at T_18 h_; this was significant at T_24 h_.

**Table 1:** Haemodynamic variables

	Group	T_baseline_	T_ALI_	T_6 h_	T_18 h_	T_24 h_
Heart rate (beats per minute)	PCV	72 (66–85)	73 (67–76)	93 (74–110)	79 (74–87)	81 (69–90)
	PCV+R	71 (60–85)	89 (73–100)	92 (77–101)	91 (78–101)	85 (66–93)
Mean arterial pressure (torr)	PCV	69 (60–79)	83 (72–93)	84 (65–99)	68 (59–73)	67 (49–77)
	PCV+R	72 (69–80)	93 (81–101)^a,b^	72 (70–73)^c^	61 (56–65)^b-d^	55 (51–59)^b-d^
Central venous pressure (torr)	PCV	7 (6–7)	7 (6–7)	7 (5–9)	7 (6–8)	8 (7–9)
	PCV+R	4 (3–6)	7 (6–9)^b^	8 (5–11)^b^	8 (7–9)^b^	8 (8–9)^b^
Cardiac output (litres per minute)	PCV	4 (3.7–4.9)	4.8 (4.7–5)	5.1 (4.5–5.7)	3.9 (3.5–4.6)	3 (2.8–3.7)^c,d^
	PCV+R	4.8 (3.7–6.5)	6.8 (5.4–8.1)^a,b^	4 (3.6–4.2)^a,c^	3.8 (3.5–4.7)^c^	3.9 (3.3–4.3)^c^

Data are presented as the median (interquartile range). ^a^Mann-Whitney rank sum test: *p *< 0.05 versus PCV. ^b-d^Friedman repeated measures analysis of variance on ranks with all pairwise multiple comparison procedures (Student-Newman-Keuls method): ^b^versus T_baseline_, ^c^versus T_ALI_, ^d^versus T_6 h _(*p *< 0.05). PCV, pressure-controlled ventilation; PCV+R, pressure-controlled ventilation with recruitment manoeuvre; T_6 h_, time point 6 hours after randomisation; T_18 h_, time point 18 hours after randomisation; T_24 h_, time point 24 hours after randomisation; T_ALI_, time point after lung injury; T_baseline_, baseline time point.

Variables of gas exchange are shown in Table 2. The PaO_2_/FiO_2 _index was comparable in the two groups after the induction of lung injury at T_ALI_. After randomisation, the PaO_2_/FiO_2 _index was improved in the PCV+R group more than fourfold and remained constant during the trial. The difference from the PCV group was significant at T_6 h_. In the PCV group, the oxygenation did improve in a number of animals, but there was no significant increase over time. Hypercapnia developed in both study groups during the induction of lung injury and became worse in the PCV group until T_6 h_. A statistically significant difference in PaCO_2 _was present at the end of the experiment at T_24 h_.

**Table 2:** Gas exchange

	Group	T_baseline_	T_ALI_	T_6 h_	T_18 h_	T_24 h_
PaO_2_/FiO_2 _(torr)	PCV	592 (572–603)	70 (59–71)^a^	62 (50–217)^a^	120 (69–315)^a^	208 (78–340)^a^
	PCV+R	554 (519–569)^b^	56 (52–63)^a^	260 (245–278)^a-c^	240 (227–265)^a,c^	250 (244–269)^a,c^
PaCO_2 _(torr)	PCV	39 (35–46)	49 (41–58)^a^	62 (56–68)^a,c^	61 (50–64)^a,c^	64 (57–67)^a,c^
	PCV+R	39 (37–42)	55 (50–71)^a^	54 (40–64)^a^	47 (44–62)^a^	48 (45–54)^a,b^

Data are presented as the median (interquartile range). ^a^Friedman repeated measures analysis of variance on ranks with all pairwise multiple comparison procedures (Student-Newman-Keuls method): versus T_baseline _(*p *< 0.05). ^b^Mann-Whitney rank sum test: *p *< 0.05 versus PCV. ^c^Friedman repeated measures analysis of variance on ranks with all pairwise multiple comparison procedures (Student-Newman-Keuls method): versus T_ALI _(*p *< 0.05). FiO_2_, inspiratory fraction of oxygen; PaCO_2_, arterial carbon dioxide tension; PaO_2_, arterial oxygen tension; PCV, pressure-controlled ventilation; PCV+R, pressure-controlled ventilation with recruitment manoeuvre; T_6 h_, time point 6 hours after randomisation; T_18 h_, time point 18 hours after randomisation; T_24 h_, time point 24 hours after randomisation; T_ALI_, time point after lung injury; T_baseline_, baseline time point.

Serum enzymes (Table 3) showed a more than fivefold and eightfold increase of AST within 24 hours of ventilation after T_ALI _in the PCV and PCV+R groups, respectively. Similarly, LDH increased more markedly during the trial after T_ALI _in the PCV+R group and reached twice the values of the PCV group. In contrast, the liver-specific enzymes ALT and GGT were not elevated compared to baseline values, nor were AP values. Liver synthesis, measured by ChE, remained stable after T_ALI_. Bilirubin levels were low, but it has to be mentioned that in the PCV+R group bilirubin levels increased after T_ALI _and were twice those of the PCV group. These changes in total bilirubin were attributable to direct bilirubin. Elevated lactate levels were noticeable at baseline and slightly higher in the PCV+R group after T_ALI_.

**Table 3:** Liver serum markers

	Group	T_baseline_	T_ALI_	T_6 h_	T_18 h_	T_24 h_
AST, U/l	PCV	26 (24–30)	29 (27–32)^a^	50 (42–58)^a,b^	81 (69–155)^a-c^	150 (103–191)^a-d^
	PCV+R	31 (25–38)	29 (28–37)	126 (64–231)^a,b^	138 (105–197)^a,b^	262 (230–364)^a-e^
ALT, U/l	PCV	31.2 (24.8–35.5)	28.1 (25.4–29.9)^a^	26 (25.1–28.7)^a^	25.6 (20.2–31.6)^a^	23.5 (20.1–30)^a^
	PCV+R	39.3 (36–44.7)	36.3 (31.5–41)	37.6 (34.6–40.7)^e^	32.5 (30.5–36.8)	37.3 (33.8–37.8)
GGT, U/l	PCV	42.5 (37.6–43.2)	35.7 (32.9–38.6)^a^	37.6 (31.9–40.6)^a^	36.5 (31–38.4)^a^	34.3 (29.1–34.7)^a^
	PCV+R	37.8 (34.3–42.7)	38.5 (30–41.6)	34.6 (28.4–38.2)^a,b^	33.6 (27.8–36.6)^a,b^	33.3 (27–35.6)^a,b^
LDH, U/l	PCV	544 (482–585)	498 (442–545)	550 (492–606)^a,b^	587 (565–814)^a,b^	647 (580–921)^a,b^
	PCV+R	607 (437–716)	565 (474–799)	677 (645–1,039)^a,b^	1,012 (653–1,689)^a-c^	1,262 (872–2,082)^a-e^
AP, U/l	PCV	99 (71–122)	91 (64–123)^a^	92 (70–113)^a^	93 (65–106)^a^	84 (60–110)^a-d^
	PCV+R	82 (79–94)	78 (76–89)	88 (82–98)	87 (82–93)	86 (78–90)
ChE, U/l	PCV	356 (339–471)	357 (307–422)	387 (315–433)	363 (323–403)	318 (297–417)
	PCV+R	431 (359–461)	381 (353–437)^a^	413 (333–425)^a^	358 (339–429)^a^	368 (339–423)^a^
TotBili, μmol/l	PCV	3.4 (3.0–5.1)	1.7 (1.7–2.1)	2.6 (1.7–3.4)	3.4 (1.7–5.1)	3.4 (1.7–3.8)
	PCV+R	4.3 (3.4–7.3)	3.4 (3.0–4.3)^a^	6.8 (6.0–9.0)^a,b,e^	6.8 (4.7–7.3)^a,b^	8.6 (6.4–9.4)^a,b,e^
DirBili, μmol/l	PCV	0.8 (0.5–0.9)	0.3 (0.2–0.5)	0.4 (0.3–0.7)	0.6 (0.3–1.0)	0.6 (0.3–1.0)
	PCV+R	0.7 (0.5–0.9)	0.7 (0.5–1.1)^e^	1.4 (0.6–1.8)^e^	1.6 (1.3–1.8)^e^	2.2 (1.7–2.3)^e^
Lactate, mmol/l	PCV	4.2 (2.9–10.4)	1 (1–1.6)^a^	0.7 (0.7–0.9)^a,b^	0.7 (0.7–0.8)^a,b^	0.7 (0.5–0.9)^a,b^
	PCV+R	4 (3.2–6.8)	0.9 (0.8–1.1)^a^	1.3 (1.2–2.1)^a,b,e^	0.9 (0.8–1)^a,c^	1 (0.9–1.2)^a,c,e^

Data are presented as the median (interquartile range). ^a-d^Friedman repeated measures analysis of variance on rank with all pairwise multiple comparison procedures (Student-Newman-Keuls method). ^a^versus T_baseline_, ^b^versus T_ALI_, ^c^versus T_6 h_, ^d^versus T_18 h _(*p *< 0.05). ^e^Mann-Whitney rank sum test: *p *< 0.05 versus PCV. ALT, alanine aminotransferase; AP, alkaline phosphatase; AST, aspartate aminotransferase; ChE, cholinesterase; DirBili, direct bilirubin GGT, γ-glutamyltransferase; LDH, lactate dehydrogenase; PCV, pressure-controlled ventilation; PCV+R, pressure-controlled ventilation with recruitment manoeuvre; T_6 h_, time point 6 hours after randomisation; T_18 h_, time point 18 hours after randomisation; T_24 h_, time point 24 hours after randomisation; T_ALI_, time point after lung injury; T_baseline_, baseline time point; TotBili, total bilirubin.

**Figure 2:**
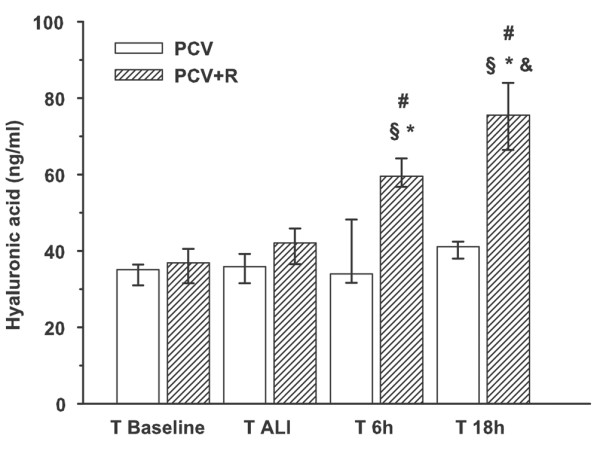
Hyaluronic acid serum concentration. Data are presented as the median with 25% to 75% interquartile range. Mann-Whitney rank sum test: #, *p *< 0.05 versus PCV+R. Friedman repeated measures analysis of variance on ranks with all pairwise multiple comparison procedures (Student-Newman-Keuls method): §, versus T_baseline_; *, versus T_ALI_; &, versus six hours (*p *< 0.05). PCV, pressure-controlled ventilation; PCV+R, pressure-controlled ventilation with recruitment manoeuvre; T_6 h_, time point 6 hours after randomisation; T_18 h_, time point 18 hours after randomisation; T_ALI_, time point after lung injury; T_baseline_, baseline time point.

Degradation of ICG, shown as plasma disappearance rate, was moderately increased at all time points compared to baseline; this increase was not statistically significant if the blood clearance of ICG was evaluated (Table 4). There was no impairment of ICG metabolisation after T_ALI_. HA (Figure 2) showed an accumulation after T_ALI _in the PCV+R group, and median serum levels increased from 42 (interquartile range 37 to 46) to 75 (66 to 84) ng/ml until the end of the experiment, whereas the concentrations in the PCV group remained stable. There was no correlation between the plasma disappearance rate or blood clearance of ICG and the serum levels of HA at any examined time point (data not shown).

**Table 4:** Metabolisation of indocyanine green

	Group	T_baseline_	T_ALI_	T_6 h_	T_18 h_
PDR (%/minute)	PCV	9.7 (9.1–10.3)	11.3 (10.7–11.6)^a^	16.9 (13.2–18.2)^a,b^	14.7 (13.3–16.1)^a,b^
	PCV+R	11.8 (10.6–12.9)	14.4 (12.5–17.4)^a,c^	15.4 (12.1–17.1)^a^	18.3 (13.6–20.7)^a^
CB (ml/minute)	PCV	322 (283–365)	421 (393–455)	512 (406–567)	477 (458–496)
	PCV+R	477 (419–540)^c^	536 (456–664)	489 (461–510)	550 (469–625)

Data are presented as the median (interquartile range). ^a,b^Friedman repeated measures analysis of variance on rank with all pairwise multiple comparison procedures (Student-Newman-Keuls method): ^a^versus T_baseline_, ^b^versus T_ALI _(*p *< 0.05). ^c^Mann-Whitney rank sum test: *p *< 0.05 versus PCV+R. CB, blood clearance of indocyanine green; PCV, pressure-controlled ventilation; PCV+R, pressure-controlled ventilation with recruitment manoeuvre; PDR, plasma disappearance rate of indocyanine green; T_6 h_, time point 6 hours after randomisation; T_18 h_, time point 18 hours after randomisation; T_ALI_, time point after lung injury; T_baseline_, baseline time point.

**Figure 3:**
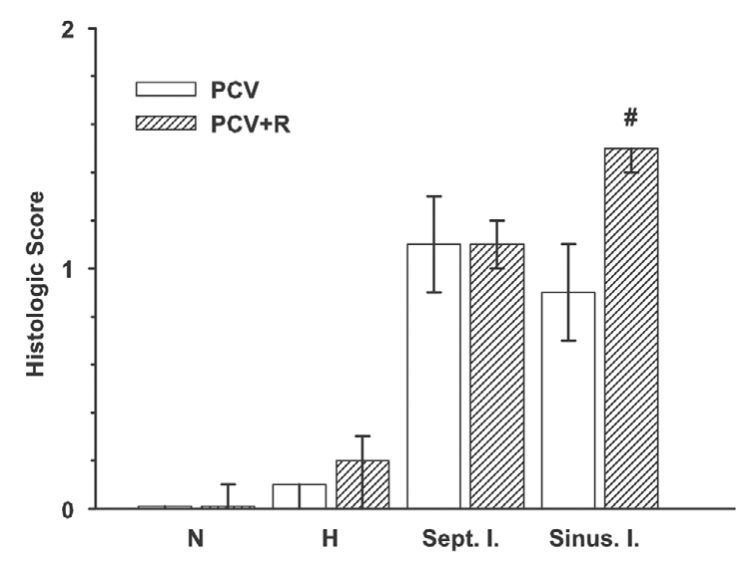
Histologic injury score of liver tissue. Scores were calculated as follows: injury not present (grade 0), less than or equal to 20% of the lobulus (grade 1), more than 20% to less than or equal to 50% (grade 2), or more than 50% (grade 3). The mean score for every animal was calculated out of 10 evaluations. Data are presented as the median with 25% to 75% interquartile range. Mann-Whitney rank sum test: #, *p *< 0.05 versus PCV+R (*p *< 0.05). H, haemorrhage; N, necrosis; PCV, pressure-controlled ventilation; PCV+R, pressure-controlled ventilation with recruitment manoeuvre; Sept. I., septal inflammation; Sinus. I., sinusoidal inflammation.

**Figure 4:**
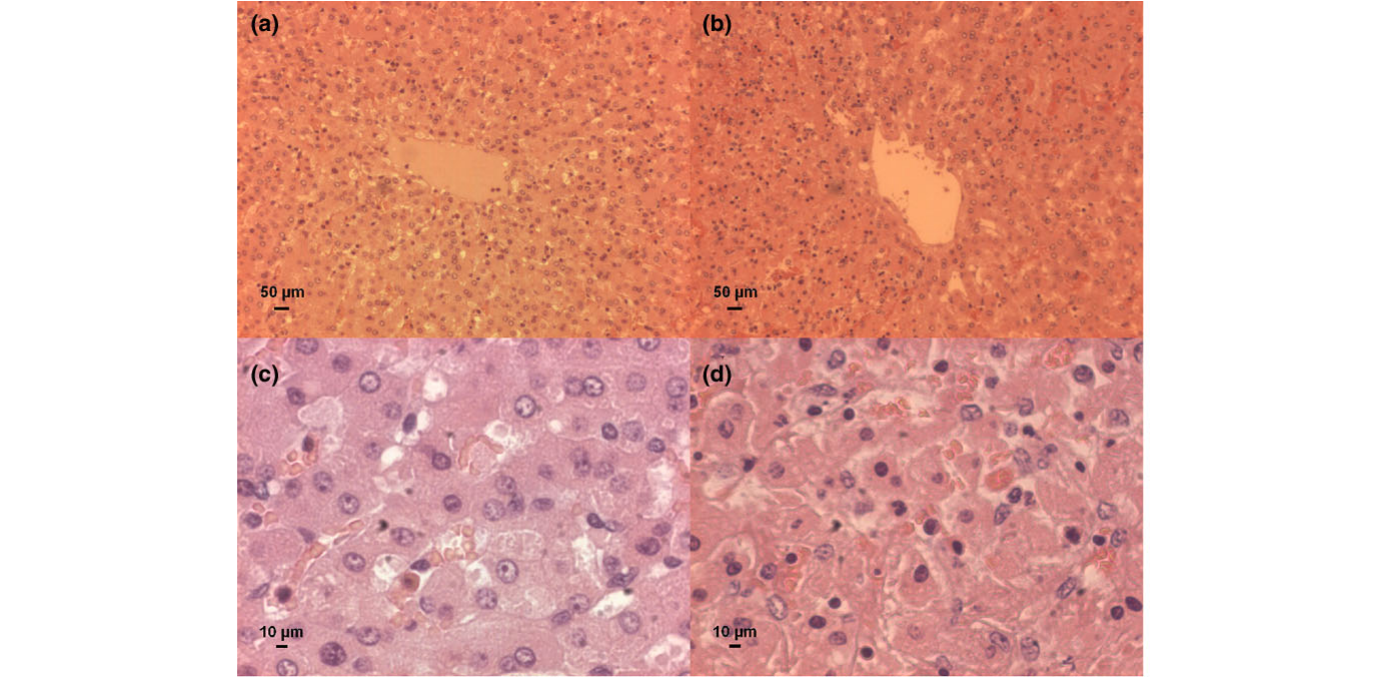
Accumulation of inflammatory cells in liver sinusoids. Representative centrolobular parts of liver tissue sections stained with haematoxylin and eosin are shown as low-power fields **(a,b) **and high-power fields **(c,d) **of the same area (optical magnification of microscope × 10 and × 40). **(a,c) **Animal from the PCV group. Few inflammatory cells are shown. **(b,d) **Animal from the PCV+R group. Infiltration of polymorphonuclear neutrophils and lymphocytes in the sinusoidal area of the liver is shown. PCV, pressure-controlled ventilation; PCV+R, pressure-controlled ventilation with recruitment manoeuvre.

Histological examination of the liver (Figure 3) showed no obvious differences in necrosis, haemorrhage, or portal inflammation, but the grading of sinusoidal inflammation was moderately higher in the PCV+R group (*p *= 0.01), with a median grade (interquartile range) of 1.5 (1.4 to 1.5) compared to 0.9 (0.7 to 1.1) in the PCV group. There was a good correlation of the histological evaluation of both investigators (*r *= 0.88; *p *< 0.001) for all four parameters together. To quantify the inflammatory reaction within the sinusoidal area of the liver, polymorphonuclear neutrophils and lymphocytes were counted. The median number of neutrophils in 10 hpf was higher in PCV+R animals: 27 (14.5 to 36.5) versus 9 (6.5 to 10) (*p *< 0.001) in the PCV group. In addition, more lymphocytes were found in the PCV+R group: 27.5 (23.5 to 33.5) versus 10.5 (9.5 to 11.5) (*p *= 0.003) in the PCV group. Figure 4 illustrates the findings in the liver sinusoids.

## Discussion

This study examined liver injury dependent on two different ventilatory approaches. One strategy used low PEEP levels throughout; the other used a recruitment manoeuvre with high PEEP levels in the beginning, followed by adaptation of PEEP to avoid hypoxaemia. We found a more prominent inflammatory reaction in the liver sinusoids of animals with recruitment during the trial. Accordingly, hyaluronan levels and other serum markers of liver injury (LDH, AST, lactate, and so on) were increased in this group. Recruitment was associated with liver damage connected with inflammation. Elevated serum levels of HA may indicate impaired uptake by damage to the endothelium of the liver sinusoids or increased release because of degradation of matrix hyaluronan in the liver.

To our knowledge, this is the first study to examine liver pathology dependent on airway pressures. Valenza and colleagues [16] tested the influence of different tidal volumes with a constant mean airway pressure on the pathology of rat liver. They found a trend toward more sinusoidal oedema without inflammatory changes. A similar ventilation protocol was used by Imai and colleagues [3] to study apoptosis in lung, kidney, small intestine, and liver in rabbits. The injurious ventilation, consisting of high tidal volumes and low PEEP, led to more apoptotic cells in the kidney and small intestine and less apoptotic cells in the lung; no increased apoptosis of liver cells in either group was detected [3].

A number of animal studies have shown that the application of increasing levels of PEEP reduces hepatic arterial and portal venous blood flow in proportion to the decrease in cardiac output [17,18]. The reduction of hepatic blood flow, at least up to certain PEEP levels, can be reversed by volume replacement restoring cardiac output [5]. In the present study, extra colloid solution was administered before recruitment, but finally colloid volume was not different in the two groups. Cardiac output was not a controlled factor and was depressed at higher PEEP levels during the PEEP trial but was comparable between the two groups afterward. Thus, liver perfusion was presumably decreased during recruitment.

Sha and colleagues [18] showed that intrahepatic oxygen delivery was impaired more than expected by applying a PEEP of 20 cm H_2_O than by reduced hepatic blood flow alone because portal venous oxygen content was already decreased. Nevertheless, hepatic oxygen consumption was not significantly diminished due to increased hepatic oxygen extraction. In our study, hepatic blood flow and hepatic venous oxygen content were not measured, but even higher levels of PEEP were applied in animals already having a disturbed gas exchange after induction of lung failure. Therefore, the ability of the liver to compensate oxygen demand by a higher extraction ratio may have been exhausted. Beyer and colleagues [19] measured a decreased liver tissue PaO_2 _with 20 cm H_2_O of PEEP. Thus, in our experiment, a deficiency in oxygen supply may account for the histopathologic findings in the centrolobular region of the liver receiving the least oxygenated blood. An additional pathogenic factor could be increased sinusoidal pressures given that liver congestion can already be found in animals treated with a PEEP of 10 cm H_2_O [17], most probably induced by elevation of the hepatic venous pressure. Only a single sample of liver tissue was taken in our experiment. This was from the ventral part of the liver. Eventually, samples from dorsal parts of the liver which are exposed to higher hydrostatic pressures might exhibit different and even more prominent damage. However, the influences of the different macro-haemodynamic forces like hepatic arterial and portal venous flow on the hepatic microcirculation and concomitantly on hepatic injury in the context of mechanical ventilation in our model were not the focus of this study and await further investigation.

Besides direct effects of ventilation on the liver, prolonged recruitment may liberate mediators, causing endothelial damage in the liver sinusoidal cells, or may lead to inflammatory cell activation. Imai and colleagues [3] postulated such remote organ injury by serum factors given that plasma from rabbits treated with an injurious ventilation method induced more *in vitro *apoptosis in cultured renal proximal tubular cells.

The findings in liver histopathology were accompanied by an increase of HA levels in the PCV+R group only. Similarly, bilirubin was slightly elevated in this group. Serum AST and LDH increased in both groups, but this was more marked in the group with recruitment manoeuvre. AST is highly concentrated in liver cells and is elevated in hepatobiliary diseases but is not specific [20]. Centrolobular damage found in the histologic examination can also be found in toxic and ischaemic hepatitis, which is more relevant for our model. Accordingly, elevations of AST are found in ischaemic hepatitis [21], but here ALT is also elevated, and furthermore no relevant necrosis was present in our liver samples. Another possibility is an impaired clearance of AST liberated elsewhere given that AST is endocytosed by liver sinusoidal cells [22].

The clearance of ICG was slightly increased from baseline values after induction of lung injury paralleled by cardiac output but was not reduced after induction of lung injury at the chosen points of measurement. Measurement of ICG clearance is mainly influenced by liver blood flow, because in doses up to 0.5 mg/kg hepatic extraction rate is high in the absence of severe liver damage. Therefore, Bonnet and colleagues [23] used the ICG clearance method for the determination of hepatic blood flow during application of PEEP in patients while they presumed a constant fraction of ICG absorbed by the liver dependent on hepatic blood flow. They found a reduction of hepatic blood flow by PEEP which correlated with cardiac output. However, it is understandable that ICG clearance was not different between the two groups after recruitment given that cardiac output was similar and that liver damage did not involve the hepatocytes, which take up the dye and excrete it into the bile [24].

Liver extraction of HA was improved in pigs one hour after reperfusion following liver transplantation if *S*-adenosyl-l-methionine, a protective agent against ischaemia-reperfusion injury, was administered [25]. Accordingly, sinusoidal congestion and dilatation, centrilobular necrosis, endotheliitis, and sinusoidal infiltration by polymorphonuclear cells and lymphocytes were mitigated. HA uptake is impaired by decreased liver blood flow to a certain extent. In our experiments, liver blood flow was not measured, but the kinetics of HA over time makes such an influence unlikely. HA levels increased over time in one group, whereas the difference in mean airway pressures possibly impairing liver blood flow became smaller until the end of the experiment. Accordingly, differences in cardiac output between the two groups were no longer significant at T_18 h_. We presume that sinusoidal endothelial cell damage is induced during the recruitment manoeuvre, but cell function worsened during the following ventilation period.

HA and HA degradation products themselves are thought to positively contribute to innate immunity as well as to acute inflammation via different receptor interactions (CD44, toll-like receptors) in macrophages during acute lung injury [10,11]. Proinflammatory stimuli upregulate HA expression on endothelial cells [26]. Endothelial cells mediate neutrophil [27] and lymphocyte [28] adhesion and transendothelial emigration via a CD44/HA interaction. Therefore, increased production of hyaluronan in the liver or other organs besides decreased clearance could be another explanation for elevated serum hyaluronan. Hallgren and colleagues [29] detected approximately 30-fold higher levels of serum HA in patients with ARDS for extrapulmonary causes compared to patients before minor surgery with general anaesthesia. Here, concentrations of HA in bronchoalveolar lavage fluid were also elevated and were related to the severity of the disease, but differences from control patients were not so pronounced compared with serum results. Nevertheless, bronchoalveolar lavage concentrations of albumin were also very high. Therefore, the authors concluded that blood HA was not the source of lung HA. However, all animals in our experiment had lavage-induced ARDS, but serum HA remained stable at low baseline levels during the ventilation in the PCV group. Histologic examination of the lung damage score was not different between the two groups in our experiment (data not shown). Nevertheless, it has to be recognised that our results may not be transferable to ARDS with a different pathogenesis. The purpose of this study was to examine liver injury occurring with different ventilation strategies in lavage-induced ARDS. Values of different markers, including HA serum levels, were examined to detect liver dysfunction in this setting. We have been able to show that HA serum levels as well as AST, LDH, and bilirubin were in concordance with the detected histological results. However, the pathophysiological role of HA and its degradation products in our model have to be further investigated.

## Conclusion

The animal group treated with lung recruitment showed an accumulation of neutrophils and lymphocytes in the sinusoidal area of the liver. This group had increased serum liver enzyme and HA concentrations six hours after recruitment, whereas animals without recruitment showed no changes in their liver enzyme and HA levels until the last measurement at 18 hours. Because the endothelial cells in the sinusoidal part of the liver (which showed the inflammatory reaction) degrade HA, we strongly presume that the two events are linked. Therefore, elevated levels of HA may be an early indicator for the detection of liver damage elicited by ventilation strategies with recruitment manoeuvres and sustained inflation in acute lung injury. Whether the liver damage is a consequence of changes in liver perfusion or remote effects of lung recruitment has to be addressed in further trials.

## Key messages

• The treatment of experimental ARDS with pronounced high-pressure ventilation implicated liver inflammation, but plasma disappearance rate of ICG indicated no impaired liver function.

• Animals treated with PCV exhibited significantly less inflammatory cells in liver sinusoids than animals treated with PCV and a recruitment manoeuvre (PCV+R).

• HA and liver enzyme levels in the PCV+R group were significantly higher than in the PCV group.

• Elevated levels of HA can be an early indicator for the detection of liver damage in experimental ARDS.

## Abbreviations

ALT = alanine aminotransferase; AP = alkaline phosphatase; ARDS = Acute respiratory distress syndrome; AST = aspartate aminotransferase; ChE = cholinesterase; FiO_2 _= inspiratory fraction of oxygen; GGT = γ-glutamyltransferase; HA = hyaluronic acid; hpf = high-power fields; ICG = indocyanine green; LDH = lactate dehydrogenase; PaCO_2 _= arterial carbon dioxide tension; PaO_2 _= arterial oxygen tension; PCV = pressure-controlled ventilation; PCV+R = pressure-controlled ventilation with recruitment manoeuvre; PEEP = positive end-expiratory pressure; T_6 h_, time point 6 hours after randomisation; T_12 h_, time point 12 hours after randomisation; T_18 h_, time point 18 hours after randomisation; T_24 h_, time point 24 hours after randomisation; T_ALI_, time point after lung injury; T_baseline_, baseline time point.

## Competing interests

The authors declare that they have no competing interests.

## Authors' contributions

MK collected data and did histological examinations, analysed the data, drafted the manuscript, and performed the statistical analysis. RMM designed the study and collected data. RWB and JB drafted the manuscript and participated in the design of the study. H-HW collected data. NR participated in the design of the study. CW performed HA assays and histological examinations and helped interpret the results and write the manuscript. All authors read and approved the final manuscript.
